# Melatonin prevents allergic airway inflammation in epicutaneously sensitized mice

**DOI:** 10.1042/BSR20210398

**Published:** 2021-09-22

**Authors:** Xudong Liu, Yuchao Zhang, Yaolin Ren, Jinquan Li

**Affiliations:** 1 Department of Food Science and Engineering, Moutai Institute, Renhuai 564507, China; 2 School of Medicine, Wuhan University of Science and Technology, Wuhan 430065, China

**Keywords:** Allergic asthma, Atopic dermatitis, Atopic March, Oxidative stress

## Abstract

**Purpose:** The pathological process of atopic dermatitis (AD) progressing into other types of allergic diseases such as asthma and allergic rhinitis during the first several years of life is often referred to as the atopic march. Although the phenomenon of atopic march has been recognized for decades, how asthma stems from AD is still not fully understood, confounding a universal strategy to effectively protect people from the atopic march. **Methods:** We established experimental atopic march mice by first inducing allergic dermatitis with 0.5% fluorescein isothiocyante (FITC) applied to the skin, followed by an ovalbumin (OVA) airway challenge. In addition, by examining serum immunoglobulin (Ig) concentrations, airway cytokines, the levels of oxidative stress markers, histopathological changes in lung tissue and airway hyperresponsiveness (AHR), we were able to validate the successful establishment of the model. Furthermore, by detecting the attenuating effects of melatonin (MT) and the levels of oxidative stress in the atopic march mice, we explored the potential molecular mechanisms involved in the development of atopic march. **Results:** By successfully establishing an experimental atopic march mouse model, we were able to demonstrate that overproduction of oxidative stress in the lung significantly up-regulated the activation of nuclear factor-κB (NF-κB) signaling pathways causing thymic stromal lymphopoietin (TSLP) release, which further promotes the development of atopic march. **Conclusions:** To mitigate the development of the atopic march, antioxidants such as MT may be imperative to inhibit NF-κB activation in the lung, especially after the onset of AD.

## Introduction

Atopic diseases have been on the rise in recent decades, but no single risk factor can sufficiently explain the heightened prevalence across the globe [1]. There is robust evidence suggesting that the characteristic sequence of atopic manifestations typically occurs first with atopic dermatitis (AD) in infancy, followed by allergic rhinitis and/or asthma in later stages. In addition, the severity of AD appears to influence the course of respiratory allergy in adulthood [2]. The pathological process of AD progressing into other types of allergic diseases such as asthma and allergic rhinitis during the first several years of life is often referred to as the atopic march [3]. Both AD and asthma share similar atopy phenotypes, which include T helper type 2 inflammation with eosinophilia, and hyper-IgE immunoglobulinemia [4]. However, the molecular mechanisms underlying the atopic march, and how asthma stems from AD, remains obscure. Thus, further research is needed to understand the development of these disorders and to find interventions for mediating the occurrence of atopic diseases.

Excessive levels of reactive oxygen species (ROS) and other oxidation products may result in oxidative damage, an important mechanism for the pathophysiology of allergic diseases including AD and asthma [5]. In particular, alveolar epithelial type II cells, a type of lung cell, are especially susceptible to the effects of oxidants [6]. Along with a significant increase in ROS, pro-inflammatory cytokines (tumor necrosis factor-α (TNF-α) and IL-1β) were found in BEAS-2B cells treated with PM_2.5_ [7]. Moreover, excessive production of ROS can initiate inflammatory responses by activating redox-sensitive transcription factors, such as nuclear factor-κB (NF-κB), activator protein-1 (AP-1), and hypoxia-inducible factor (HIF)-1 [8–10]. Various studies have reported the importance of the NF-κB pathway in promoting the production of cytokines during allergic disease pathogenesis [11]. These studies suggest that assessment of oxidative stress levels and activation of the NF-κB pathway, may be a promising approach for understanding the development of atopic diseases.

Thymic stromal lymphopoietin (TSLP), an airway epithelium-derived cytokine, plays a central role in polarizing dendritic cells (DCs) and induces the differentiation of naive T cells into Th2 cells [12]. Lung fibroblasts and bronchial smooth muscle cells can also produce TSLP under inflammatory conditions initiated by IL-13 stimulation [13]. Previous studies have suggested the critical role of keratinocytic TSLP in the development of AD into asthma [14,15]. However, few studies have pinpointed the specific role of TSLP during the asthmatic phase of atopic march, let alone its mechanism of activation. In allergic asthma, TSLP activates DCs and promotes the homeostasis shift of Th1/Th2 into Th2, which subsequently results in airway remodeling and sustained airway hyperresponsiveness (AHR) [16]. Blocking or knocking out TSLP or its receptor (TSLPR) in mice can prevent the development of airway inflammation and hyperactivity in response to exogenous antigens [17]. It has been reported that the human TSLP gene promoter contains binding sites for, and can be regulated by NF-κB [18]. In addition, ROS triggers the Th2 immune response by promoting the production of oxidized lipids, which are responsible for the Toll-like receptor 4 (TLR4)-mediated induction of TSLP in epithelial cells [19].

Melatonin (MT), a major hormone released by the pineal gland mostly at night, is a receptor-independent free-radical scavenger with potent antioxidant properties in organs [20]. Previous studies showed that MT treatment not only suppresses the production of ROS but also decreases the level of lipid peroxidation. Furthermore, MT increases the release of several antioxidant enzymes, including glutathione peroxidase (GPx), glutathione reductase (GR) and superoxide dismutase (SOD), indicating that MT plays a protective role in redox balance and cell damage [21,22]. It has been shown that the administration of MT protects against exogenous substance-aggravated allergic asthma [23], however, the effect and mechanism underlying the protection by MT in an experimental atopic march model is poorly understood. Therefore, determining the levels of oxidative stress, NF-κB and TSLP in the lung tissue of the experimental atopic march mice after MT administration, may help to provide a better understanding of the development of the atopic march.

In the present study, we established experimental atopic march mice by first inducing allergic dermatitis to achieve 0.5% FITC, followed by an ovalbumin (OVA) airway challenge. In addition, by examining serum immunoglobulin concentrations, airway cytokines, histopathological changes in lung tissue and AHR, we were able to validate the successful establishment of the model. Furthermore, by determining the attenuating effects of MT and the levels of oxidative stress in atopic march mice, explored the potential molecular mechanisms involved in the development of atopic march.

## Materials and methods

### Animals

Hubei Province Experimental Animal Center (Wuhan, China) provided male Balb/c mice (4–5 weeks, 20 ± 1.5 g) and the commercial diet for the present study. All the mice were housed in the animal experimental center of Wuhan University of Science and Technology, subject to a 12-h light–dark cycle with *ad libitum* access to water and food. All animal experiments took place at the Animal Experimental Center of Wuhan University of Science and Technology following relevant guidelines and regulations. The Office of Scientific Research Management of Wuhan University of Science and Technology approved the experimental protocol, with approval ID: WUST-IACUC-201814. Mice were killed by cervical dislocation under pentobarbital sodium anesthesia.

### Main reagents and kits

Acetone, dibutyl phthalate (DBP, 99%), fluorescein isothiocyante (FITC), MT and OVA were obtained from Sigma–Aldrich (St. Louis, MO, U.S.A.). All other chemicals were of analytical grade. Mouse enzyme-linked immunosorbent assay (ELISA) kits for total IgE were obtained from Biolegend (San Diego, CA, U.S.A.), and OVA-IgE and OVA-IgG1 were purchased from BlueGene (Shanghai, China). ELISA kits for IL-1β, TNF-α, IL-4, IL-5, IL-13, interferon (IFN)-γ, IL-33 and TSLP were all obtained from eBioscience (San Diego, CA, U.S.A.). The glutathione (GSH) and malonaldehyde (MDA) test kits were obtained from Nanjing Jiancheng Bioengineering Institute (Nanjing, Jiangsu, China). The protein test kit was provided by Sangon Biotech (Beijing, China).

### Experimental design and procedure

A 0.5% FITC-mediated contact hypersensitivity (CHS) is a Th2-dominant immune system and elevated levels of IL-4 expression in the inflamed skin and increased IgE levels in the serum. Because AD shares many features with CHS to FITC, this model of Th2-type CHS was used in the present study to induce AD-like skin lesions and then aerosol challenge of OVA to establish the mice model of atopic march.

**Figure 1:**
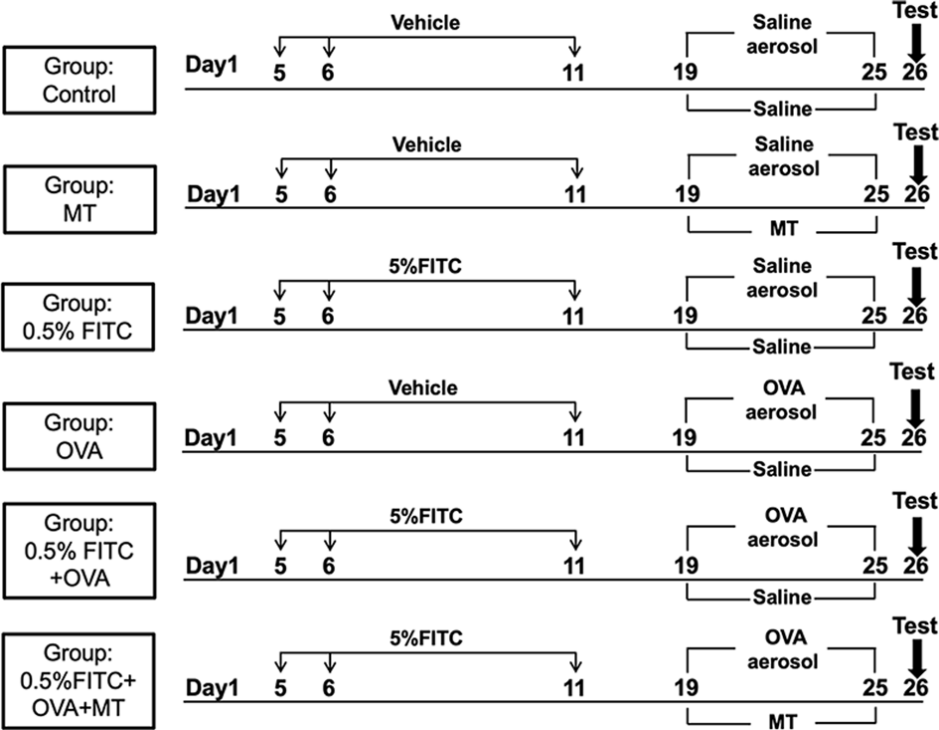
Exposure and sensitization protocol

There are six groups in this experiment with six mice per group. (1) Control group (Control) mice were treated with 120 μl of vehicle (1:1 DBP/acetone) on their shaven backs on days 5 and 6. On day 11, their shaven backs and left ears were subjected to 40 μl of vehicle. In addition, the mice were exposed to an aerosol challenge of saline (30 min/day) using an ultrasonic nebulizer (Yuyue 402A type I, China) on days 19 through 25. (2) MT control group (MT) mice received the same treatment as the control group, but in addition the mice were treated with 5 mg/(kg.day) MT by intratracheal instillation, from days 19 to 25. (3) A 0.5% FITC-induced AD group (0.5% FITC) mice were sensitized with 120 μl of 0.5% FITC on days 5 and 6 on their shaven backs, and on day 11 their shaven backs and left ear were subjected to 40 μl of 0.5% FITC [24]. From days 19 to 25, the mice were exposed to saline (30 min/day) using an ultrasonic nebulizer. (4) OVA exposure group (OVA) mice were exposed to an aerosol challenge of 1% OVA (30 min/day) using an ultrasonic nebulizer on days 19 through 25. (5) A 0.5% FITC and OVA co-treatment group (0.5% FITC+OVA) mice were treated with 120 μl of 0.5% FITC on days 5 and 6 on their shaven backs, and on day 11, their shaven backs and left ear were subjected to 40 μl of 0.5% FITC. The mice were then challenged with 1% OVA using an ultrasonic nebulizer from days 19 to 25. (6) A 0.5% FITC and MT exposure combined with OVA challenged group (0.5% FITC+OVA+MT). These mice were treated with 120 μl of 0.5% FITC on days 5 and 6 on their shaven backs, and on day 11 their shaven backs and left ear were subjected to 40 μl of 0.5% FITC. From days 19 to 25, these mice were challenged with 1% OVA using an ultrasonic nebulizer. In addition, 5 mg/(kg.day) MT was administered by intratracheal instillation from days 19 to 25. The detailed protocol is shown in Figure 1.

### Quantitative analyses of total serum IgE, OVA-IgE, and OVA-IgG1

Heart blood was collected after the mice were anesthetized with pentobarbital on day 18. Immediately after collection, the blood serum was centrifuged at 3000 rpm for 10 min at room temperature. Serum total-IgE, OVA- IgE and OVA- IgG1 levels were measured using commercial ELISA kits according to the manufacturer’s instructions.

### Assessment of IL-1β and TNF-α

Serum levels of IL-1β and TNF-α were measured using commercial ELISA kits according to manufacturer’s protocols. The sensitivity of the kit was 5 pg/ml.

### Analysis of cytokines and inflammatory cells in bronchoalveolar lavage fluid

After serum collection, the mice were killed. The trachea was exposed for alveolar lavage, by cutting the neck skin. Sterile saline (0.9 ml) was injected through the trachea into the lungs and sucked out after a 1-min chest massage. This procedure was repeated three times, and the bronchoalveolar lavage fluid (BALF) centrifuged at 1500 rpm at 4°C for 10 min. The supernatant was collected for measuring the levels of IL-4, IL-5, IL-13, IFN-γ, IL-33 and TSLP in the lung using commercial ELISA kits. In addition, the cell sediment from the centrifuged BALF was suspended in saline, and total inflammatory cells, neutrophils and eosinophils were counted using a Blood Cell Analysis system (MTN-21, Matee3nu Technology Corp, Jinan, China).

### Histology

The left lungs of the mice were removed and inflated with 4% paraformaldehyde, fixed overnight at 4°C and embedded in paraffin. Sections (5 μm) were stained using Hematoxylin and Eosin (H&E). Briefly, the sections on slides were deparaffinized and rehydrated with distilled water and kept in Hematoxylin for 1 min. After washing five-times, the slides were counterstained in Alcoholic-Eosin for 1 min. The slides were then dehydrated with 95% EtOH and 100% EtOH, followed by the application of Xylene to clear for 1 min before the coverslip was mounted. Masson’s Trichrome staining was used to show the peribronchial collagen deposition. Lung slides were incubated with mordant (50 mg/ml potassium bichromate + 50 mg/ml trichloroacetic acid) for 20 min and then washed for 1 min. Next, the slides were put into Weigert’s iron Hematoxylin solution for 3 min, washed with water for 10 min and then incubated with mordant (2.5% phosphomolybdic acid + 2.5% phosphotungstic acid) for 30 s. Following 1-min wash, the slides were incubated in 1% acetic acid for 10 s and then 0.75% Orange G solution for 5 min. Before and after staining with Ponceau xylidine (12 mg/ml) and Fuchsin S solution (8 mg/ml) for 30 min, the sections were washed with 1% acetic acid for 10 s, and then incubated with 2.5% phosphotungstic acid followed by Aniline Blue solution for 3 min. After washing with 1% acetic acid for 10 s, the sections were dehydrated with a graded ethanol series (70, 80 90, 95, and 100%), defatted with xylene twice and mounted. Periodic Acid–Schiff (PAS) staining reveals the increased mucus production that occurs after allergen exposure due to goblet cell hyperplasia. The lung section slides were placed in a staining dish and fixed with Carnoy’s fixative for 10 min. After three washes, the slides were stained with periodic acid solution for 10 min. Before and after Shiff Reagent staining, the slides must be rinsed very carefully. The solutions were dehydrated in ascending alcohol and cleared with xylene in a staining dish, and then the coverslip was mounted. All sections of the lung tissue were assessed by pathologists in a blinded fashion using a DM4000B microscope.

### Assessment of AHR

AHR was determined using a lung function system (AniRes2005 version 2.0, Beijing, China) on day 26. Briefly, experimental mice were anesthetized by intraperitoneal injection of pentobarbital sodium (100 mg/kg). The trachea was exposed and cannulated, and then attached to a computer-controlled ventilator. The ventilator set the standard parameters to be 1:1.5 ratio of inspiration/breath and 90 breaths per minute. Four doses of methacholine (MCH; 0.025, 0.05, 0.1 and 0.2 mg/kg), a constrictor agonist, were injected into the jugular vein at 5-min intervals. Any change in R-area of the inspiratory resistance (Ri) and expiratory resistance (Re) and in the value of pulmonary dynamic compliance (Cldyn) were recorded within 250 s after the MCH injection. The severity of AHR is represented by increased R-area of Ri, Re and a decrease in Cldyn.

### Lung tissue homogenate preparation and GSH and MDA determination

After washing the lung tissue in ice-cold phosphate-buffered saline (PBS), 10% of the lung tissue homogenate samples were prepared by homogenizing on ice using 10 ml/g of ice-cold PBS (pH 7.5). The homogenate was centrifuged at 12000 rpm for 10 min at 4°C, and the supernatant collected for GSH and MDA testing. The total protein in each sample was measured using a modified BCA protein assay kit. The concentrations of GSH and MDA were determined in accordance with the kit’s manufacturer’s instructions.

### Immunohistochemical staining of TSLP and NF-κB (phospho-S536)

Sections were blocked in 5% normal goat serum and incubated with the primary antibodies anti-TSLP (1:100, Abcam, MA, U.S.A.) and anti-phospho-p65 (S536) (1:100, Abcam, MA, U.S.A.), respectively. The sections were then incubated in an appropriate biotinylated immunoglobulin and avidin–biotin peroxidase complex. The negative control was obtained by omitting the primary antibody. Stained sections were observed using a DM4000B Microscope. The results of TSLP and NF-κB staining were estimated as an average of optical density (AOD) using ImagePro-Plus 6.0 software.

### Statistical analyses

The data are presented as the mean ± standard deviation. The statistical graphs were generated using GraphPad Prism 5.0 (San Diego, CA, U.S.A.). Results were evaluated statistically using a one-way analysis of variance (ANOVA) followed by Holm–Sidak’s multiple comparisons test. *P*<0.05 was considered to be a significant difference.

## Results

### Serum immunoglobulin (total IgE, OVA-IgE and OVA- IgG1) and pro-inflammatory cytokines levels in an experimental atopic march model

**Figure 2:**
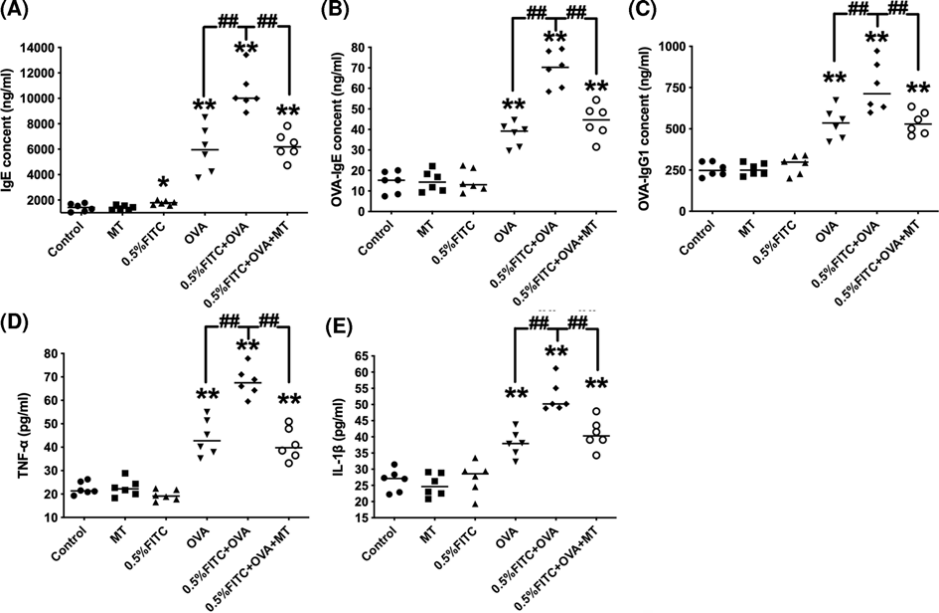
The changes in immunoglobulin levels and pro-inflammatory cytokines in the serum of atopic march mice, and the attenuating effects of MT Total IgE (**A**), OVA-IgE (**B**), OVA-IgG1 (**C**), TNF-α (**D**) and IL-1β (**E**) levels in serum. *: *P*<0.05, **: *P*<0.01, compared with the control group; ^##^: *P*<0.01, compared with the 0.5% FITC+OVA group.

For this experiment, we needed a mouse model exhibiting the atopic march features. To establish this model, the mice were first induced with AD by applying 0.5% FITC to the skin, followed by an OVA sensitization and challenge phase to induce an asthmatic phenotype. All of the OVA-sensitized groups displayed an up-regulation of total IgE, OVA-IgE and OVA-IgG1 levels compared with the control group. As expected, the experimental atopic march mice (0.5% FITC+OVA) demonstrated further elevation in serum total IgE, OVA-IgE and OVA- IgG1 levels in comparison to the OVA alone group (Figure 2A–C, *P*<0.01). The levels of pro-inflammatory cytokines, including TNF-α and IL-1β, in the OVA group were significantly higher than that of the control group (Figure 2D,E, *P*<0.01). Moreover, the concentrations of IL-1β and TNF-α exhibited exacerbated effects in the experimental atopic march group (*P*<0.01). As shown in Figure 2, treatment with MT dramatically reduced the levels of total IgE, OVA-IgE, OVA-IgG1, IL-1β and TNF-α compared with the 0.5% FITC+OVA group (*P*<0.01).

### Pro-inflammatory cytokine levels in serum, Th cytokines and the number of eosinophils in BALF measured in the experimental atopic march model

**Figure 3:**
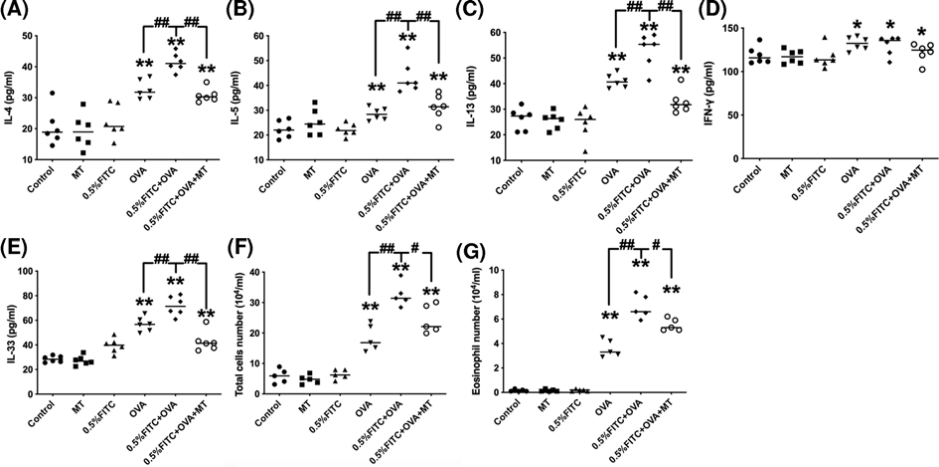
Effect of MT on cytokine levels and eosinophil numbers in BALF of atopic march mice (**A**) Levels of IL-4. (**B**) Levels of IL-5. (**C**) Levels of IL-13. (**D**) Levels of IFN-γ. (**E**) Levels of IL-33. (**F**) Number of total eosinophils (**G**) *: *P*<0.05, **: *P*<0.01, compared with the control group; ^#^: *P*<0.05, ^##^: *P*<0.01, compared with the 0.5% FITC+OVA group.

Levels of pro-inflammatory cytokines in serum, concentrations of Th2 cytokines (including IL-4, IL-5 and IL-13) and the number of eosinophils in BALF, of the experimental atopic march group, were much greater than those found in the OVA only group (Figure 3A–C,G; *P*<0.01). On the other hand, the expression levels of IFN-γ were not significantly affected in the atopic march group compared with the OVA group (Figure 3), suggesting that these mice exhibited an enhanced Th2 airway response. Moreover, the concertation of IL-33 in the 0.5% FITC+OVA group increased in comparison to the OVA group (Figure 3E; *P*<0.01). Interestingly, the concentrations of IL-4, IL-5, IL-13, IL-33 and eosinophil numbers were substantially lowered in the MT and 0.5% FITC+OVA groups compared with the atopic march group (*P*<0.01).

**Figure 4:**
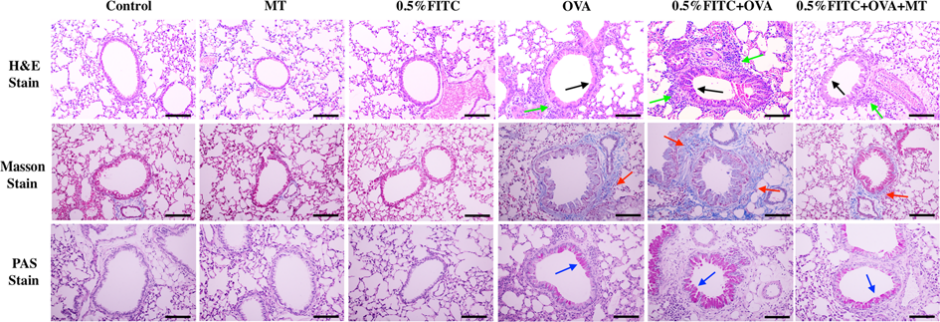
MT treatment reversed the pathological alterations in atopic march mouse model Green arrow, lung tissue cell infiltration; black arrow, bronchial remodeling; red arrow, subepithelial collagen deposition (blue colored stain); blue arrow, mucus hypersecretion (pale pink colored stain), scale bar = 50 μm.

H&E staining, Masson’s staining and PAS staining (Figure 4) were carried out to evaluate any pathological alterations in the experimental atopic march model. Compared with the OVA only group, the atopic march group demonstrated significantly greater inflammatory cell infiltration into the peribronchial areas (green arrow) and thickening of the airway lumen (black arrow), as detected using H&E staining. Similarly, Masson’s staining showed that the lung tissue of mice in the atopic march group appeared to have a thicker subepithelial collagen layer (blue color, blue arrow) and PAS staining showed an increase in mucus secretion ability (blue arrow) as compared with the OVA only group. Moreover, co-treatment with MT in the 0.5% FITC+OVA group markedly attenuated the degree of inflammatory cell infiltration, mucus overproduction, lung fibrosis and goblet cell hyperplasia.

### AHR changes in the experimental atopic march model

**Figure 5:**
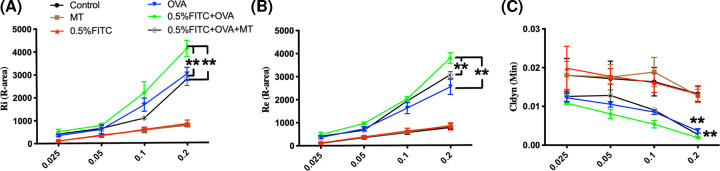
MT treatment attenuated AHR in the atopic march mouse model (**A–C**) Ri, Re and Cldyn values, respectively. **: *P*<0.01, compared with the 0.5% FITC+OVA group.

AHR, a hallmark of asthma, can be determined using Re, Ri and Cldyn after MCH challenge tests. A larger R-area under the dose–response curve after MCH treatment represents a greater maximal response, and the change of Ri and Re observed here represents the change in the large airways. These together with the lowest value of Cldyn which reveals the state of the parenchyma and small airways provides a quantitative assessment of AHR. In an effort to show the effect of atopic march on AHR in response to MCH, we demonstrated a significant increase in Ri and Re, as well as markedly reduced Cldyn in the atopic march group compared with the OVA alone group (Figure 5A–C, respectively), suggesting that the atopic march group had more severe AHR. In addition, MT treatment in the 0.5% FITC+OVA group led to significantly lower Ri and Re, as well as higher Cldyn levels compared with the 0.5% FITC+OVA group (*P*<0.01), showing that MT treatment improved the aggravated AHR in the atopic march group.

### The levels of TSLP of lung tissue in an experimental atopic march model

**Figure 6:**
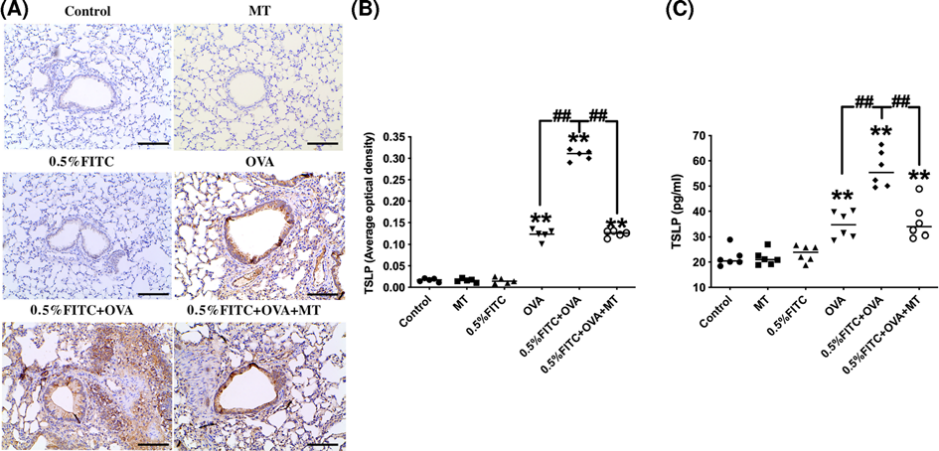
Increased production of TSLP in the lungs of atopic march mice and the attenuating effects of MT (**A**) Immunohistochemistry for TSLP in lung tissue, scale bar = 50 μm. (**B**) The optical density of immunohistochemistry for TSLP. (**C**) Concentrations of TSLP in BALF as determined by ELISA. **: *P*<0.01, compared with the control group; ##: *P*<0.01, compared with the 0.5% FITC+OVA group.

To further explicate the molecular mechanism behind the development of the atopic march, we examined the expression of TSLP using both immunohistochemistry (Figure 6A) and ELISA (Figure 6C). Average optical density was used to represent the level of TSLP protein detected using immunohistochemistry (Figure 6B). As shown in Figure 6, the levels of TSLP in the OVA group were significantly greater than that of the control group (*P*<0.01). In addition, compared with the OVA only group, the expression of TSLP was markedly increased in the experimental atopic march group (*P*<0.01). The expression of TSLP was lower in the 0.5% FITC+OVA+MT group than in the 0.5% FITC+OVA group (*P*<0.01).

### The levels of oxidative stress and NF-κB in an experimental atopic march model

**Figure 7:**
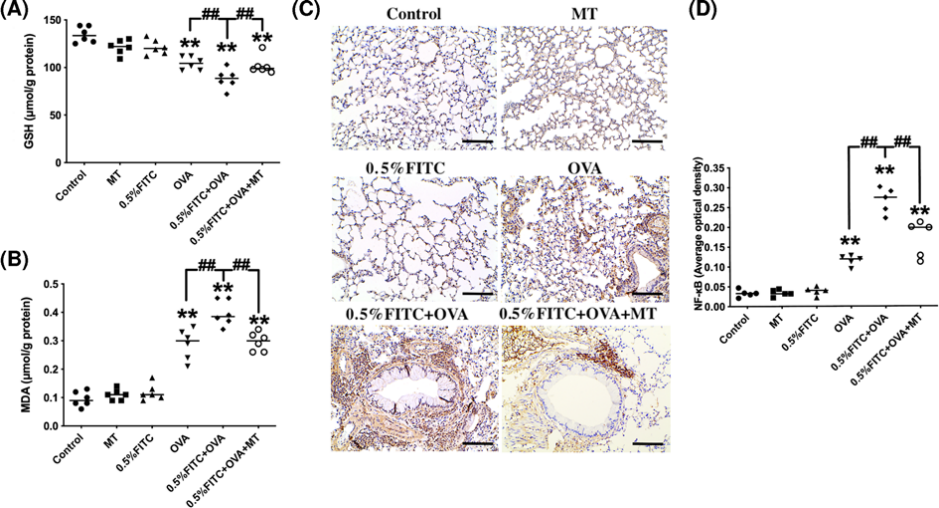
MT treatment decreased the levels of oxidative stress and the change of activated NF-κB content in the atopic march mouse model Concentrations of GSH (**A**) and MDA (**B**) in the lung. (**C**) Immunohistochemistry for NF-κB in lung tissue, scale bar = 50 μm. (**D**) The optical density of immunohistochemistry for NF-κB. **: *P*<0.01, compared with the control group; ^##^: *P*<0.01, compared with the 0.5% FITC+OVA group.

GSH and MDA were measured in the lung to evaluate the level of oxidative damage in the experimental atopic march model. In comparison with the control group, the OVA group demonstrated significantly lowered levels of GSH (*P*<0.05) and extremely significantly increased levels of MDA (*P*<0.01). The GSH levels in the atopic march mice were lower than those of the OVA group (Figure 7A). The MDA levels in the atopic march group were extremely significantly increased compared with the OVA group (Figure 7B). The immunohistochemistry results of NF-κB staining are shown in Figure 7C, estimated as the average optical density using Image-Pro Plus 6.0 software (Figure 7D). The concentration of activated NF-κB was enhanced in the OVA group (*P*<0.01) compared with the control group, and the levels of activated NF-κB were further increased in the atopic march group compared with the OVA group (*P*<0.01). The presence of MT attenuated the increase in MDA, and the decrease in GSH and activated NF-κB levels seen in the 0.5% FITC+OVA group (*P*<0.01).

## Discussion

Understanding the mechanism behind the development of asthma triggered by skin sensitization, is crucial for elucidating the underlying mechanism of the atopic march, as this information could indicate possible therapeutic preventions. In the present study, we demonstrate that after skin sensitization with 0.5% FITC, allergic asthma symptoms induced by OVA were significantly increased. In addition, high levels of oxidative stress and TSLP were also detected in lung tissues. We conclude that the overproduction of oxidative stress markers and TSLP may be an important aspect in AD-related asthma.

We established an experimental mouse model representing atopic march features. In contrast with previous studies which only focus on the role of barrier-impaired skin, we tried to explore the possible conditions for aggravated asthmatic symptoms after the induction of AD lesions. The atopic march mouse model was established by first treating the skin with 0.5% FITC, followed by an OVA airway challenge to induce asthmatic symptoms. We did not induce AD and asthma at the same time, so as to mimic a more natural physiological process for exogenous allergen sensitization, in contrast with previous sensitization methods [25,26]. AD is initially driven by sensitization to environmental antigens due to altered epidermal barrier functioning of the skin, which can subsequently lead to immune response activation [27]. Results from this study and our previous studies suggest that, after sensitization and challenge with 0.5% FITC, changes in immunological and inflammatory biomarkers (total immunoglobulin (Ig) E, as well as histopathological examination and measurement of ear swelling (Supplementary Figure S1) in our CHS model), were all consistent with other studies [28,29]. These results demonstrate that 5% FITC sensitization elicits AD-like skin lesions in this mouse model. Aggravated symptoms of OVA-induced asthma in response to previous allergic dermatitis, signals the success of the atopic march model. To investigate the progression of AD to respiratory allergies, an OVA-sensitized mouse asthma model was established in the present study. The degree to which AD had progressed to asthma was evaluated by looking at three typical features of allergic asthma: airway inflammation; airway remodeling and AHR. Th2 lymphocytes contribute to the pathogenic progression of asthma and to the concentrations of T-IgE, OVA-IgE and OVA-IgG1, which are crucial for a clinical diagnosis of allergic asthma [30]. In this study, when compared with the OVA group, the atopic march group had significantly raised levels of T-IgE, OVA-IgE, OVA-IgG1, IL-1β and TNF-α in serum as well as IL-4, IL-5 and IL-13 in BALF (Figures 2 and 3). These results suggest that in comparison to the OVA-induced mice, the experimental atopic march model mice demonstrated an exacerbated inflammation response. In addition, the added effects of AD on OVA-induced allergic asthma were also confirmed by lung histopathological changes and AHR [31]. More serious airway remolding, worsened inflammatory cell infiltration, increased mucus secretion and increased AHR, were all observed in the atopic march mice (Figures 4 and 5). Based on these results, we conclude that AD may lead to worsened OVA-induced asthma symptoms.

Oxidative stress is a crucial factor in the pathophysiology of allergic asthma. Previous studies have provided a potential link between increased oxidative damage and the development, or worsening of allergic asthma [32]. In this study, exacerbated allergic asthma symptoms were found in the atopic march group compared with the OVA group, accompanied by the induction of pulmonary tissue oxidative stress, as indicated by decreased GSH and increased MDA levels (Figure 7A,B). The decreased GSH and increased MDA levels are responsible for airway inflammation [33], and may contribute to the pathogenesis of the atopic march. It is well known that NF-κB pathways are activated by oxidative stress, which can then participate in inflammation regulation [34]. Consistent with oxidative stress detection, the level of NF-κB in the lung was exacerbated in the atopic march group compared with the OVA group (Figure 7C,D).

As a key switch in triggering Th2 responses in allergic asthma, TSLP anchors are typically induced/promoted/activated by damaged epithelial cells [35]. TSLP can directly act on CD4^+^ T cells to initiate exogenous allergens, and leading to the development of allergic inflammation [36]. Excess TSLP can also activate DCs to promote the recruitment of more Th2 cells to sites of tissue lesions, which will result in more Th2 cytokines and total/specific IgE and IgG1 production [37]. Notably, there is evidence that the promoter of the TSLP gene contained binding sites for NF-κB, and TSLP could be regulated by NF-κB in both human and mouse airway epithelial cells [38]. Moreover, it has been reported that abnormal keratinocyte differentiation is linked to NF-κB-dependent induction of TSLP in these cells [39,40]. Our results for NF-κB and TSLP, revealed similar trends to these other studies.

MT, a free-radical scavenger, effectively blocks oxidative stress [41]. By examining the activities of alanine aminotransferase (ALT) and aspartate aminotransferase (AST) in the serum (Supplementary Figure S2) and inflammation levels, we confirmed in the present study and other studies [42], that a treatment of 5 mg/(kg.day) MT is safe because the liver enzymes AST and ALT were not affected. More future studies on its safety on vital organs and functions are important to be investigated for short and long terms. Interestingly, the asthma symptoms and oxidative stress in mice subjected to 0.5% FITC treatment and an OVA challenge can be effectively alleviated by MT treatment, suggesting that oxidative stress plays an important role in the development of the atopic march. Previous studies have already demonstrated the crucial role of TSLP in the atopic march, by promoting allergen sensitization in barrier-impaired skin [14,25]. However, not enough information is available on the role of TSLP in the lung during the atopic march, nor on the possible activation mechanisms. In this study, we found that OVA treatment activated the release of TSLP, and that Th2 inflammatory responses followed in the lung. TSLP is an important target protein for regulating the inflammatory response in the asthmatic phase of atopic march. In addition, it had been proved that MT could alleviate inflammation by inhibiting the NF-κB signal *in vivo* [43]. We found that inhibition of oxidative stress by MT effectively alleviated the activation of NF-κB signaling pathways, and decreased the production of TSLP when compared with the OVA group, which further alleviated the development of atopic march. In addition to this, further studies for evaluating the effect of MT on 0.5% FITC or OVA-induced allergic inflammation are also important to be revealed.

In conclusion, by successfully establishing an experimental atopic march mouse model, we were able to demonstrate that overproduction of oxidative stress in the lung significantly up-regulated the activation of NF-κB signaling pathways and the subsequent release of TSLP, which further promotes the development of the atopic march. From a therapeutic point of view, to mitigate the development of atopic march, antioxidants such as MT may be imperative for inhibiting NF-κB activation in the lung, especially after the occurrence of AD.

## Supplementary Material

Supplementary Figures S1-S2

## Data Availability

All the data generated or analyzed during the present study are included in this published article.

## Abbreviations

AD: atopic dermatitis
AHR: airway hyperresponsiveness
ALT: alanine aminotransferase
AST: aspartate aminotransferase
BALF: bronchoalveolar lavage fluid
CHS: contact hypersensitivity
Cldyn: pulmonary dynamic compliance
DBP: dibutyl phthalate
DC: dendritic cell
ELISA: enzyme-linked immunosorbent assay
FITC: fluorescein isothiocyante
GSH: glutathione
H&E: Hematoxylin and Eosin
IFN: interferon
Ig: immunoglobulin
MCH: methacholine
MDA: malonaldehyde
MT: melatonin
NF-κB: nuclear factor-κB
OVA: ovalbumin
PAS: Periodic Acid–Schiff
Re: inspiratory resistance
Ri: expiratory resistance
ROS: reactive oxygen species
TNF-alpha: tumor necrosis factor-α
TSLP: thymic stromal lymphopoietin
