# Supplementary material for: Melatonin prevents allergic airway inflammation in epicutaneously sensitized mice
Source: Biosci Rep. 2021 Sep 22;41(9):BSR20210398. doi: 10.1042/BSR20210398 (PMC8458693; doi:10.1042/BSR20210398)
Supplement: Supplementary Figures S1-S2 [file BSR-2021-0398_supp.pdf]

Supplementary information

Supplementary methods

1. Measurement of ear swelling and Histological examination

Ear swelling was measured as our previous study [1]. Briefly, right (R0) and left ear (L0) thickness was first measured on day 5 using a vernier caliper. And second right (R) and left ear (L) thickness values obtained on day 26. Ear edema was expressed as (R±L) ± (R0±L0). After measurement of ear swelling, the right ears of mice were collected and fixed overnight in 10% formalin at room temperature. They were then sectioned, and stained with hematoxylin and eosin. Tissue sections were observed using a DM 4000B Microscope (Leica, Berlin, Germany).

2. Measurement activitiesforalanine aminotransferase (ALT) and aspartate aminotransferase (AST)

The activities of ALT (catalog #:C009-2-1) and AST (catalog #:C010-2-1) in the serum were tested by the commercial kits from Nanjing Jiancheng Bioengineering Institute (Nanjing, China), according to the manufacturer's instructions.

Supplemental Figure S1

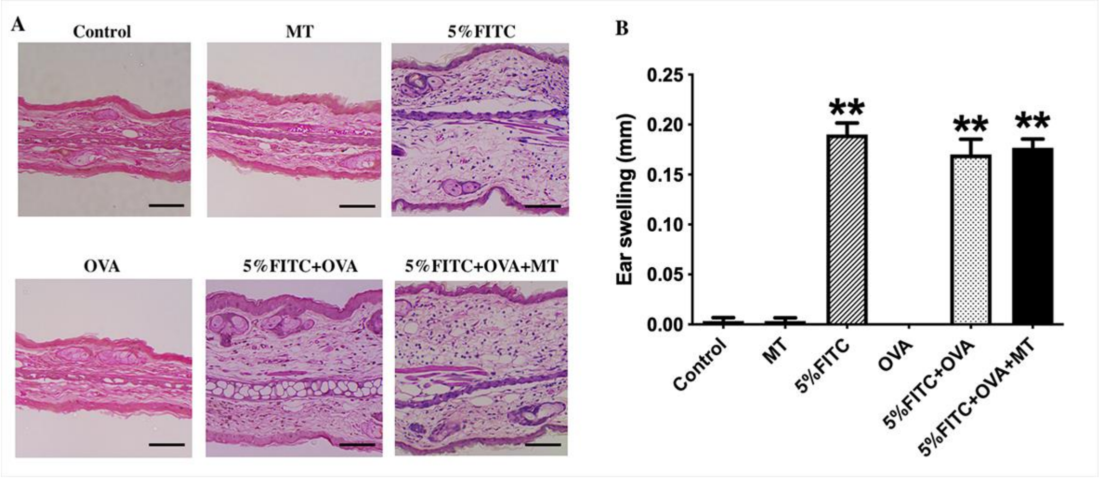

Figure S1.Measurement results of ear swellingin the atopic march mouse model. (A)Stained with hematoxylin & eosin (H&E). (B) The results of ear swelling. **: p<0.01, compared with control group.

Supplemental Figure S2

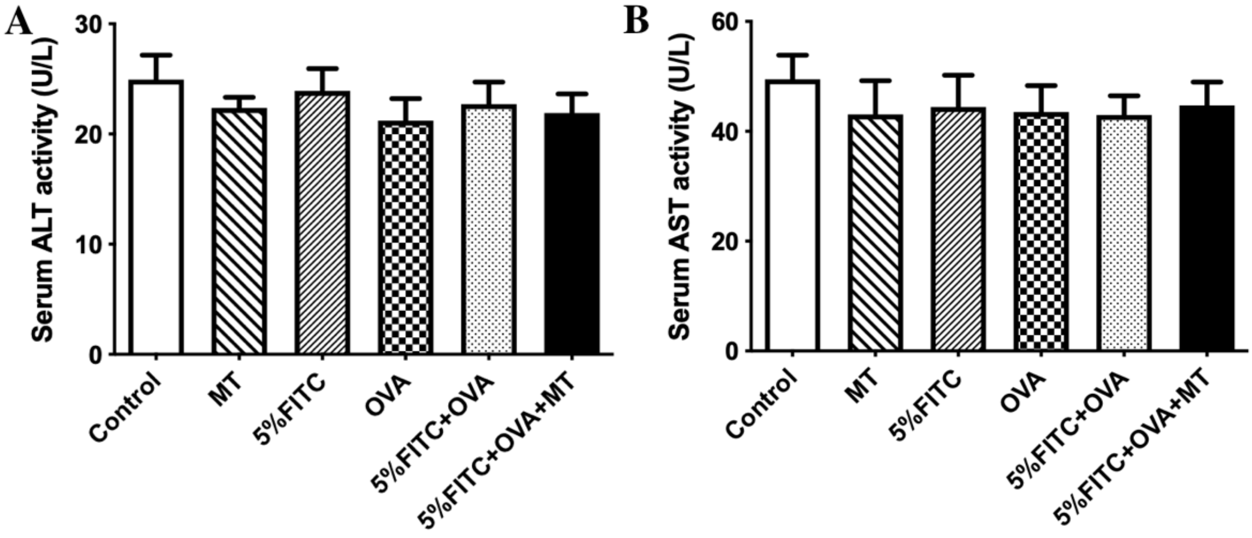

Figure S2.Effects of MT treatment on serum alanine aminotransferase (ALT) and aspartate aminotransferase (AST)in the atopic march mouse model. (A)The effect of MT on serum ALT, (B) The effect of MT on serum AST.

References

[1] Li, J., et al., 2014a. T-helper type-2 contact hypersensitivity of Balb/c mice aggravated bydibutyl phthalate via long-term dermal exposure. PloS One 9, e87887.
